# Correction: Dendrimers meet zwitterions: development of a unique antifouling nanoplatform for enhanced blood pool, lymph node and tumor CT imaging

**DOI:** 10.1039/d6nr90086k

**Published:** 2026-05-28

**Authors:** Zhijuan Xiong, Yue Wang, Jingyi Zhu, Xin Li, Yao He, Jiao Qu, Mingwu Shen, Jindong Xia, Xiangyang Shi

**Affiliations:** a State Key Laboratory for Modification of Chemical Fibers and Polymer Materials, College of Chemistry, Chemical Engineering and Biotechnology, Donghua University Shanghai 201620 People's Republic of China xshi@dhu.edu.cn; b Department of Radiology, Shanghai Songjiang District Central Hospital Shanghai 201600 People's Republic of China xiajd_21@163.com; c College of Materials Science and Engineering, Donghua University Shanghai 201620 People's Republic of China; d CQM-Centro de Química da Madeira, Universidade da Madeira, Campus da Penteada 9000-390 Funchal Portugal

## Abstract

Correction for ‘Dendrimers meet zwitterions: development of a unique antifouling nanoplatform for enhanced blood pool, lymph node and tumor CT imaging’ by Zhijuan Xiong *et al.*, *Nanoscale*, 2017, **9**, 12295–12301, https://doi.org/10.1039/C7NR03940A.

The authors regret an error in Fig. S8 in the originally published SI, where the incorrect images for the histological changes in the heart and kidney of mice at one month post intravenous injection of {(Au^0^)_100_-G5·NHAc-mPEG_20_} were provided. The SI has now been updated online with the corrected figure, as shown herein.

An independent expert has viewed the corrected figure and has concluded that it is consistent with the discussions and conclusions presented in the article.


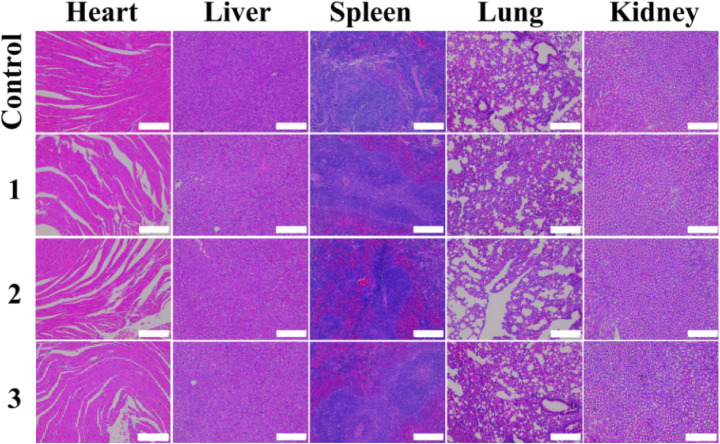
Fig. S8 Histological changes in heart, liver, spleen, lung, and kidney of the mice at one month post intravenous injection of {(Au^0^)_100_-G5·NHAc-CBAA_20_} (1), {(Au^0^)_100_-G5·NHAc-CBAA_80_} (2) or {(Au^0^)_100_-G5·NHAc-*m*PEG_20_} (3) ([Au] = 0.1 M, 150 μL in saline, for each mouse) (*n* = 3). Mice injected with PBS were used as control. These organ sections were H&E stained and observed under an optical microscope (the scale bar in each panel indicates 200 μm).

The Royal Society of Chemistry apologises for these errors and any consequent inconvenience to authors and readers.

